# Hyperpolarized Renal Magnetic Resonance Imaging: Potential and Pitfalls

**DOI:** 10.3389/fphys.2016.00072

**Published:** 2016-03-01

**Authors:** Christoffer Laustsen

**Affiliations:** Department of Clinical Medicine, MR Research Centre, Aarhus UniversityAarhus, Denmark

**Keywords:** dynamic nuclear polarization, hyperpolarization, magnetic resonance imaging, renal metabolism

## Abstract

The introduction of dissolution dynamic nuclear polarization (d-DNP) technology has enabled a new paradigm for renal imaging investigations. It allows standard magnetic resonance imaging complementary renal metabolic and functional fingerprints within seconds without the use of ionizing radiation. Increasing evidence supports its utility in preclinical research in which the real-time interrogation of metabolic turnover can aid the physiological and pathophysiological metabolic and functional effects in *ex vivo* and *in vivo* models. The method has already been translated to humans, although the clinical value of this technology is unknown. In this paper, I review the potential benefits and pitfalls associated with dissolution dynamic nuclear polarization in preclinical research and its translation to renal patients.

## Renal magnetic resonance imaging

Magnetic resonance imaging (MRI) is a harmless, non-ionizing imaging modality that provides excellent soft tissue contrast. Although this technique has been used successfully in several applications, its full potential is seldom utilized *in vivo* because of its limited sensitivity. This low sensitivity increases the acquisition times beyond acceptable time scales for detecting metabolically active molecules and substrates in low concentrations. To date, renal MRI is used primarily for morphological examinations in clinical practice. However, for renal functional imaging, several potentially important alternatives exist, which can provide information on renal physiological status in terms of fibrosis, oxygenation, and glomerular filtration. These methods have yet to be translated to clinical practice (Prasad, 2006; Notohamiprodjo et al., 2010). Preclinical and clinical studies have indicated that these methods hold promise for improving the management and outcome of patients with renal diseases (Prasad, 2006; Notohamiprodjo et al., 2010).

The complex pathophysiology of renal disease is closely associated with metabolic alterations that contribute to the disease or are caused as a result of disease progression. Despite tremendous achievements in understanding the basic mechanism of renal disease, scientists still have poor insight into the metabolic link between the development and treatment of renal disease. This is partly because the methods employed to investigate these mechanisms are often destructive *ex vivo* methods or *in vivo* radiolabeled tracer techniques.

Advances in hyperpolarization technology have opened up new avenues for increasing the sensitivity of diagnostic imaging in humans using both spin exchange optical pumping (SEOP) and dissolution dynamic nuclear polarization (d-DNP) hyperpolarization. SEOP enables hyperpolarization of noble gases such as Xenon-129 and Helium-3, while d-DNP enables hyperpolarization of carbon-13 in solution (Ardenkjaer-Larsen et al., 2003). This review focuses on d-DNP for renal imaging applications.

## Dissolution dynamic nuclear polarization

**Figure 1:**
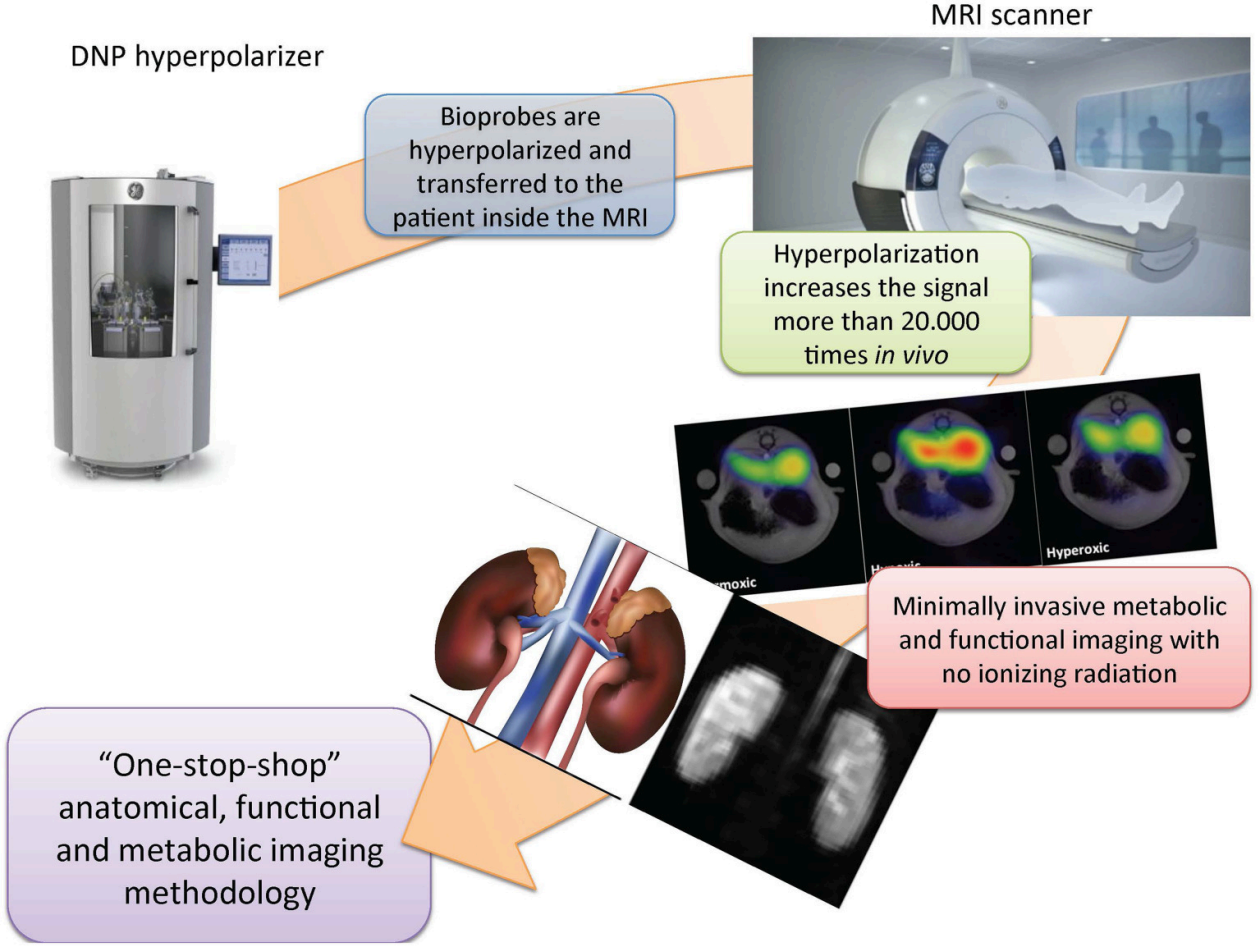
**Dissolution dynamic nuclear polarization magnetic resonance imaging—a “one-stop-shop” methodology**. The biomarker is hyperpolarized and transferred by intravenous injection to the patient inside the clinical scanner. This procedure increases the sensitivity of the measurements by more than 20,000 times, and enables the direct interrogation of renal uptake and metabolic turnover. Standard clinical magnetic resonance imaging (MRI) capabilities are simultaneously available and provide a very versatile and sensitive diagnostic modality that is free of ionizing radiation and thereby ensures patient safety. DNP, dissolution dynamic nuclear polarization.

Dissolution dynamic nuclear polarization (d-DNP) is a method that extends the already vast applicability of MRI to provide real time *in situ* cellular metabolic information (Ardenkjaer-Larsen et al., 2003, 2011). The method relies on the generation of a transient artificial high signal 10,000-fold greater than the thermal signal at room temperature at clinical MR magnetic field strengths. This is achieved by placing the sample in a high magnetic field (typically 3–5 T) at low temperature (typically 1.3–0.8 K), and irradiating it with microwaves to transfer energy from electron spins to nuclear spins (typically carbon-13), creating the hyperpolarized sample. The hyperpolarized sample is then rapidly dissolved to obtain a liquid solution retaining the transient hyperpolarized signal (Figure 1).

d-DNP MRI relies on an intravenous bolus injection of carbon-13 (^13^C)-enriched biomarkers. Thus, high renal perfusion, metabolic activity, and altered metabolic and functional status in renal diseases makes this technology useful for renal investigations (Johansson et al., 2004; Golman and Petersson, 2006; Leupold et al., 2009). Hyperpolarized biomarkers enable direct quantification of tracer movement, as visible on positron emission tomography (PET). Similar to PET, the hyperpolarized tracer, and not modulation of the surrounding tissue, is the origin of the signal, as seen on standard contrast MRI. This allows for background-free images with high temporal resolution without the use of harmful radiation. The hyperpolarized signal is typically only observable within 1–2 min after dissolution, similar to the fast decaying PET tracers. The chemical structure dictates the signal decay rate and thus the usability of a given molecule, which leads to only small molecules being candidates for d-DNP imaging *in vivo*. The use of very small molecules ensures that bio-probes such as pyruvate, urea, and fumarate are typically freely filtered by the glomerulus and reabsorbed in the proximal tubule.

Compared to existing diagnostic tools, hyperpolarized MRI has a clear advantage in that it detects the metabolic conversion of ^13^C-labeled endogenous biomarkers into metabolic derivatives within the cells *in vivo*. Thus, it detects physiological and pathophysiological changes without the need for invasive biopsies and allows characterization of the entire parenchyma over time. This factor potentially enables separate assessment of individual kidneys, the cortex, and medulla, functional heterogeneity, and focal deficits.

Renal d-DNP MR has received increased attention because it illustrates the dynamic renal status in normal and diseased kidneys in a harmless manner (Leupold et al., 2009; Clatworthy et al., 2012; Laustsen et al., 2013, 2014a,b; Reed et al., 2014). For patients at risk of developing kidney disease, progressive knowledge of *in vivo* renal substrate selection and functional alterations may help to clarify the mechanisms that cause the kidney to fail.

The potential of d-DNP MR for metabolic and functional investigations of the kidneys was recognized early on by Golman et al. using hyperpolarized renal renograms and perfusion assessment (Golman et al., 2001; Johansson et al., 2004; Golman and Petersson, 2006); more importantly, they showed that hyperpolarized [1-^13^C]pyruvate could be used for real-time metabolic imaging *in vivo*. The localization and metabolic rate of pyruvate conversion may be important for diagnosis and for monitoring treatment in renal ischemia reperfusion (Leupold et al., 2009). It can thus serve as a marker of early renal dysfunction to guide therapeutic interventions (Laustsen et al., 2013, 2014b, 2015; Keshari et al., 2015), and highlight potential targets of therapy so as to prevent progression toward chronic kidney disease. An increasing amount of evidence supports the claim of [1-^13^C] pyruvate as a renal biomarker in diabetic nephropathy and in ischemia/reperfusion injury.

Although no alterations in renal pyruvate metabolism were detected in mice with folic acid-induced acute kidney injury, early tubular necrosis can be detected using fumarate-to-malate conversion. Fumarate does not readily enter healthy cells, and therefore, its conversion is observed only when the cell membrane is permeable. Hence, only early tubular necrosis is detectable *via* a positive malate signal (Clatworthy et al., 2012). In addition to a severely deranged pyruvate metabolism profile in the early diabetic nephropathic kidney, Keshari et al. (2015) recently showed increased oxidative stress in diabetic mouse kidneys by using the novel redox sensor, hyperpolarized [1-^13^C] dehydroascorbate. The Keshari study interestingly highlighted the potential of interrogating oxidative stress modulations, which showed that angiotensin II treatment reversed the renal redox status in the diabetic kidney. A particularly interesting alternative bioprobe for renal investigations—^13^C-urea—is sensitive to the intra-renal osmolality gradient—a hallmark of tubular function. Measuring the intrarenal distribution and perfusion of urea has been demonstrated to detect alterations in the distribution between hydration and diuresis (von Morze et al., 2012). Improved relaxation (decay rate) properties are easily incorporated by utilizing [^13^C,^15^N] urea as the bioprobe. This avoids the fast relaxation of quadrupolar nitrogen 14 (^14^N), which reduces the lifetime of the hyperpolarized sample (Reed et al., 2014). Hyperpolarized urea may ultimately reveal pathological changes in the diseased kidney. Recent novel methods that utilize the relaxation contrast mechanisms of urea are able to identify increased oxygen consumption in the early diabetic kidney and during diuresis and antidiuresis with high resolution (Reed et al., 2015; Laustsen et al., 2016). Urea shows a major potential for clinical translation as a single metabolite bioprobe, and provides simple, intuitive, and quantifiable information on the renal status. These studies together highlight the potential of a conceptual new framework for future research and drug discovery for renal diseases.

## Potential and pitfalls

Growing evidence supports hyperpolarized MRI as an excellent research tool in specialized centers; however, several potential pitfalls exist for its translation into widespread use and clinical practice. Most noteworthy are the use of apparent rate constants rather than the “true” rate constants, which would require significant invasive information on the cellular distribution of enzymes, cosubstrates, pH, temperature, and the pool sizes of the substrates for the reactions. This limitation can be partly overcome by introducing an intervention in the examination, similar to the furosemide challenge in blood oxygenation level-dependent (BOLD) MRI, which promotes a change in oxygen utilization because the required energy need is halted; however, a quantifiable measure would significantly increase the impact of the methods. The acquisition and following reconstruction strategies can also significantly impact the quantification of the experiments by imposing compromises on the available information. Hyperpolarized MRI inherently spans a five-dimensional space (i.e., three spatial, one temporal, and one spectral dimension); thus, the acquisition of the transient signal (signal decay due to image acquisition and relaxation decay) is a compromise between signal availability and the information needed to answer a particular question. Several advanced methods have thus been developed for obtaining metabolic and functional information in d-DNP experiments (Cunningham et al., 2007, 2008; Leupold et al., 2009; Mayer et al., 2009; Schmidt et al., 2014). However, this factor limits the reproducibility and comparability of the results. It is also imperative to align acquisition, reconstruction, and analysis protocols to increase the impact in research and in clinical practice, including standardization of supporting information, such as oxygenation status, heart rate, and perfusion.

Non-metabolic biomarkers such as urea for renal functional imaging can be readily quantified *via* perfusion mapping similar to positron emission tomography and relaxation mapping. This allows easier translation and interpretation of the results.

Most renal investigations have been performed in rodent models, which have unipapillary kidneys, in contrast to the multipapillary human kidney. Thus, rodent metabolism is highly elevated in comparison to that of humans. This difference between the physiology of the rat and human kidneys imposes limitations on the interpretation and translatability of the results. A limited number of studies have been performed in porcine models, which resemble the human physiology and show good agreement with the findings in rodents, along with high intra-animal reproducibility (Laustsen et al., 2015). The limited resolution often utilized in rodent studies imposes significant challenges in differentiating the cortical signal from the medullary signal. This limitation is less pronounced in large animal models; however, improved resolution is still needed.

## Human translation in renal patients

A critical point is patient safety. The current clinically ready d-DNP system relies on closed sterile samples, denoted fluid paths, and a non-contact quality control system, which ensures patient safety. The general tolerability of the pyruvate injection is high, which showed no adverse events in humans in an initial human study (Nelson et al., 2013). The production utilizes stable isotopes and requires only increased capacities of the vendors, which makes the method an already affordable technology. [1-^13^C] Pyruvate is the first bioprobe in the market, but several other candidates are in the clinical pipeline such as [2-^13^C]pyruvate, [1,4-^13^C_2_]fumarate, ^13^C-urea, and [1-^13^C]lactate.

The initial human study (Nelson et al., 2013) was performed on cancer patients. However, the biomarkers and procedures are similar for renal investigations, and thereby reduce the transfer time between patient groups. Hyperpolarized MRI shows great potential for generating new and translational insights, and thereby advances the basic understanding of renal pathophysiology and improves the basal needs for treating renal disease, even without clinical translation. To realize the clinical potential of renal hyperpolarized MRI, it is essential to improve the general availability and reproducibility of the method, to generate strong evidence of its clinical utility by performing multicenter trials, and to demonstrate the warranted evidence by comparing it to gold standard methods in patients. An especially critical point in the translation of the method is the standardization of the patient with respect to hydration and metabolic status, as illustrated by the preclinical studies. This is critical to ensure reproducibility and to maximize the sensitivity to both disease and interventions.

Although d-DNP has a few but significant pitfalls, it has great potential as a medical imaging modality. Dissolution-DNP can potentially change the medical imaging paradigm by allowing a harmless, so-called “one-stop-shop” imaging methodology. In this paper, I reviewed the advantages and the pitfalls associated with dissolution dynamic nuclear polarization in preclinical research and its translation to renal patients. The findings of this review suggest that this technology may generate new and translational insights, advance the basic understanding of renal pathophysiology, and improve the treatment of renal disease, even without clinical translation.

## Author contributions

All authors listed, have made substantial, direct and intellectual contribution to the work, and approved it for publication.

## Funding

The study was supported by The Danish Research Council, The Danish Kidney Foundation, Helen and Ejnar Bjørnows Foundation.

### Conflict of interest statement

The author declares that the research was conducted in the absence of any commercial or financial relationships that could be construed as a potential conflict of interest.

## Abbreviations

d-DNP: dissolution dynamic nuclear polarization
MRI: magnetic resonance imaging.
